# *Anaplasma phagocytophilum* Ankyrin A Protein (AnkA) Enters the Nucleus Using an Importin-β-, RanGTP-Dependent Mechanism

**DOI:** 10.3389/fcimb.2022.828605

**Published:** 2022-05-26

**Authors:** Yuri Kim, Jianyang Wang, Emily G. Clemens, Dennis J. Grab, J. Stephen Dumler

**Affiliations:** ^1^ Department of Pathology, Uniformed Services University of the Health Sciences, Bethesda, MD, United States; ^2^ Henry M. Jackson Foundation for the Advancement of Military Medicine, Bethesda, MD, United States

**Keywords:** *Anaplasma phagocytophilum*, AnkA, ankyrin repeat proteins, nuclear localization signal, RaDAR

## Abstract

*Anaplasma phagocytophilum*, a tick-borne obligately intracellular bacterium of neutrophils, causes human granulocytic anaplasmosis. Ankyrin A (AnkA), an effector protein with multiple ankyrin repeats (AR) is injected via type IV-secretion into the host neutrophil to gain access to the nucleus where it modifies the epigenome to promote microbial fitness and propagation. AR proteins transported into the host cell nucleus must use at least one of two known eukaryotic pathways, the classical importin β-dependent pathway, and/or the RanGDP- and AR (ankyrin-repeat)-dependent importin β-independent (RaDAR) pathway. Truncation of the first four AnkA N-terminal ARs (AR1-4), but not other regions, prevents AnkA nuclear accumulation. To investigate the mechanism of nuclear import, we created point mutations of AnkA N-terminal ARs, predicted to interfere with RaDAR protein import, and used importazole, a specific inhibitor of the importin α/β, RanGTP-dependent pathway. Nuclear colocalization analysis shows that nuclear localization of AnkA is unaffected by single AR1-4 mutations but is significantly reduced by single mutations in consecutive ARs suggesting RaDAR protein nuclear import. However, AnkA nuclear localization was also decreased with importazole, and with GTPγS. Furthermore, *A. phagocytophilum* growth in HL-60 cells was completely suppressed with importazole, indicating that *A. phagocytophilum* propagation requires a β-importin-dependent pathway. A typical classical NLS overlapping AR4 was subsequently identified suggesting the primacy of the importin-α/β system in AnkA nuclear localization. Whether the mutational studies of putative key residues support RaDAR NLS function or simply reflect structural changes that diminish engagement of an AR-NLS-importin pathway needs to be resolved through careful structure-function studies.

## Introduction

*Anaplasma phagocytophilum* is an obligately intracellular bacterium that colonizes human neutrophil vacuoles and is the causative agent of human granulocytic anaplasmosis, an emerging immunomodulatory tick-borne disease among humans (Bakken and Dumler, 2015). *A. phagocytophilum* utilizes type IV-secretion system effector protein ankyrin A (AnkA), which gains access to the infected neutrophil nucleus where it binds DNA, to epigenetically modify chromatin structure and transcriptional programs (Park et al., 2004; Lin et al., 2007; Garcia-Garcia et al., 2009a; Garcia-Garcia et al., 2009b; Rennoll-Bankert et al., 2015; Rikihisa, 2017; Bierne and Pourpre, 2020). This protein contains approximately 1230 amino acids, for which sequences and length vary by strain. AnkA also accumulates in the nucleus of infected cells (Garcia-Garcia et al., 2009b). As the protein’s name implies, the characteristic feature of AnkA is the presence of 8-15 ankyrin repeats (ARs), depending on the strain, a structural motif found in several hundred proteins across broad classes of eukaryotes and prokaryotes, that mediate diverse functions, such as protein-protein, protein-DNA, and protein-lipid interactions (Caturegli et al., 2000; Pan et al., 2008; Jernigan and Bordenstein, 2014; Islam et al., 2018). The AR is a conserved amino acid sequence containing approximately 33 residues which is characterized by two α-helices with 8-10 residues each connected with a β-loop. AnkA interacts with host DNA at multiple genomic sites where chromatin alterations occur leading to changes in transcriptional programs (Dumler et al., 2016); however, the mechanism of how AnkA accesses the nucleus is yet to be revealed.

We previously showed that truncation of the first four N-terminal ARs prevents nuclear accumulation of AnkA, suggesting their importance in nuclear transport (Rennoll-Bankert et al., 2015). A common mechanism of large protein-facilitated nuclear transport involves binding of importin-β and RanGDP to cargo protein in the cytoplasm, allowing the protein to cross the nuclear pore complex and to be released after RanGTP/RanGDP exchange inside the nucleus (Cavazza and Vernos, 2016). The concentration gradients of RanGDP and RanGTP across the nuclear membrane are critical for nuclear shuttling of the RanGDP/RanGTP, importin-β and cargo bound to importin-α *via* classical nuclear localization sequences, and are supported by the cytoplasmic RanGTPase-activating protein (RanGAP) and nuclear Ran guanine nucleotide exchange factor (RanGEF) (Lu et al., 2021).

For a group of human ankyrin repeat proteins (ARPs), importation into nucleus occurs due to hydrophobic residues, in particular leucine, isoleucine, phenylalanine, or cysteine at the 13^th^ position of two consecutive ARs at positions that interact with and bind RanGDP by an importin-independent mechanism (RaDAR) (Lu et al., 2014). While the precise 3D structure of AnkA ARs is not yet resolved, the four N-terminal ARs within the N-terminal region that is important for nuclear accumulation of AnkA have motifs similar to those in humans that utilize importin-independent, RanGDP-dependent (RaDAR) nuclear import (Rennoll-Bankert et al., 2015). Here, we tested whether ARs of *A. phagocytophilum* AnkA have similar requirements in nuclear accumulation as for human ARPs or whether nuclear localization is importin-dependent. While mutations of the 13^th^ residues of two consecutive ARs indeed disrupts nuclear import of AnkA and requires RanGTP, nuclear localization and *A. phagocytophilum* propagation were dependent on β-importin, confirming the essential nature of the importin α/β pathway for AnkA nuclear entry and *A. phagocytophilum* propagation.

## Results

### Single AnkA Mutations of N-Terminal ARs Do Not Prevent Nuclear Accumulation

**Figure 1:**
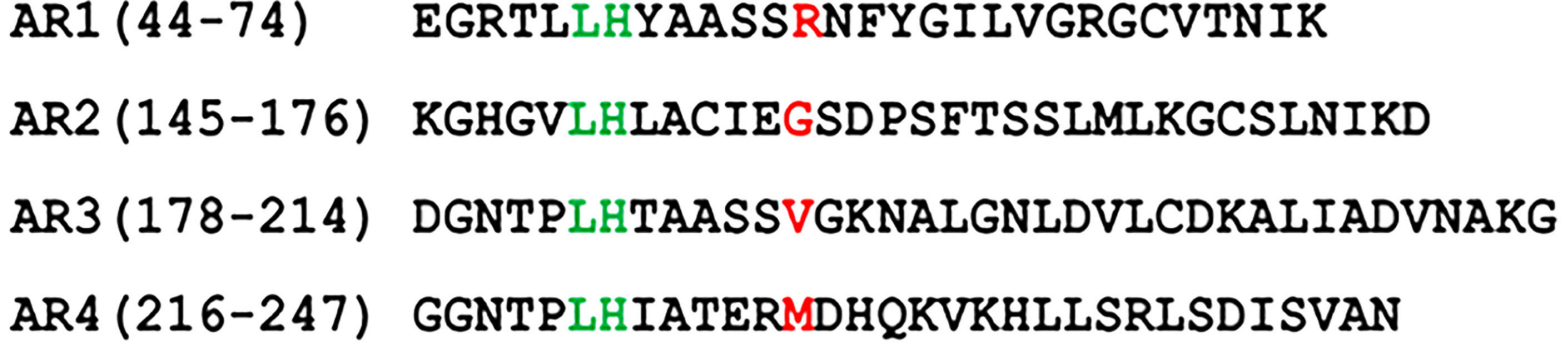
Amino acid sequences of N-terminal ankyrin repeats (AR) of *A. phagocytophilum* AnkA (WP_020849331.1). Point mutation of the amino acid at 13^th^ position tested for each AR in the study is marked in red. In AR1 the hydrophilic Arg was replaced with hydrophobic Ala (AnkA.R56A) or neutral/aliphatic Gly (AnkA.R56G). In AR2 the neutral/aliphatic Gly was replaced with hydrophobic Ala (AnkA.G157A) or hydrophilic Arg (AnkA.G157R). In AR3 the hydrophobic Val was replaced with hydrophilic Asn (AnkA.V190N) and in AR4 the hydrophobic Met was replaced with hydrophilic Arg (AnkA.M228R). Numbers in brackets show location of the corresponding ARs in full-length AnkA protein of *A. phagocytophilum* Webster^T^ strain. For proper alignment, the lysine-histidine pair conserved in all ARs is marked in green.

To test that *A. phagocytophilum* AnkA is transported into the nucleus *via* the RaDAR mechanism (Lu et al., 2014), and noting that the 13^th^ positions in AR1 and AR2 do not comply with the hydrophobic residue hypothesis, we used recombinant eGFP-tagged AnkA proteins with single mutations to alter hydrophobicity at the 13^th^ position of N-terminal ARs 1, 2, 3 and 4 (**Figure 1** and **Supplementary Figure 1**).

**Figure 2:**
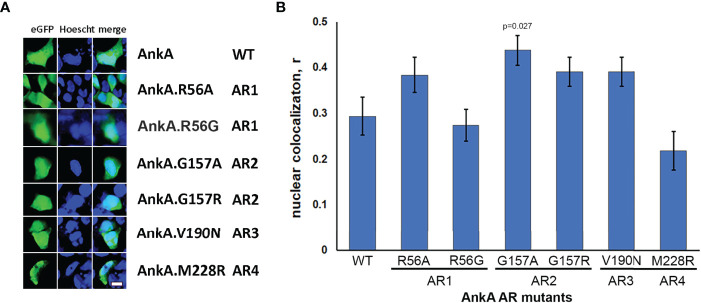
Single mutations of N-terminal ankyrin repeats did not reduce nuclear localization of AnkA. HEK293 cells were transfected with eGFP plasmid constructs of wild-type AnkA or AnkA N-terminal ankyrin repeats 1, 2, 3 and 4 with point mutations, as described in Experimental Procedures. After 48 hours, the cells were fixed, washed, stained with cell-permeable nuclear label Hoescht33342 (Hoescht) and visualized with fluorescent microscopy. **(A)** Representative images of cells transfected with eGFP-AnkA and ankyrin repeat mutants of eGFP-AnkA. **(B)** Statistical analysis of nuclear colocalization of AnkA mutants with nuclear staining relative to wild type AnkA. Data are mean ± SEM, n=20-40 of randomly selected ROIs (region of interest) containing 120-200 transfected cells. ANOVA *post hoc* Bonferroni corrected p values <0.05 relative to wild type AnkA are shown. Bar – 20 µm.

Compared to wild type AnkA, none of single mutations at the 13^th^ position of ARs 1-4 reduced AnkA nuclear localization, while nuclear localization for the AR4 AnkA.M228R mutant was significantly lower than AnkA.R56A (p=0.049), and either AnkA.G157A (p=<0.001) or G157R (p<0.031), as well as AnkA.V190N (p=0.031) (**Figure 2**). Interestingly, when the non-polar, achiral hydrophobic amino acid glycine at position 13 in AR2 was replaced with alanine, a more hydrophobic residue (AnkA.G157A), a significant increase in nuclear localization of AnkA was observed. None of the single mutants was predicted to create any structural change sufficient to damage protein function (**Supplementary Figure 2**). The unpredictable behavior on nuclear localization with these unintentional residue changes at seemingly non-critical AR positions suggests the importance of preserving the structural integrity of the N-terminal ARs. This unintentional discovery resulted when several mutants created in preliminary studies were examined for impact on nuclear localization, but after sequencing were discovered to have incorrect residue changes, substitutions or inadvertent additional mutations (**Supplementary Table 1**). Although nuclear localization studies were conducted using these mutants prior to sequence analyses, sequencing did not identify any of the proposed single mutations in this group. However, double mutations were identified to include AnkA.[R56A]; [T162A] (positions 13 in AR1 and 18 in AR2, respectively), which resulted in reduced nuclear localization, and in AnkA.[N57S]; [T162A] (position 14 in AR1 and position 18 in AR2, respectively), which did not localize to the nucleus. Similarly, mutations in non-sequential ARs 1 and 3, AnkA.[R56A (position 13 in AR1)]; AnkA.[R56A];[L183I] (positions 13 in AR1 and 6 in AR3) did not alter nuclear localization, but the non-sequential mutant that included positions 14 in AR1 and 13 in AR4 (AnkA.[N57S];[M228A] and N57S) did localize to the nucleus. Two other sequential mutants AnkA.[N57S]; [T162A]; [V190A] (positions AR1 14, AR2 18, and AR3 13, respectively), and AnkA.[T162A]; [V190A] reduced nuclear localization (**Supplementary Table 1**). Because of the inaccurate placement of these mutations for assessment of RaDAR function, none were used for primary analyses.

To investigate the potential structural impact of the individual mutations on the putative binding regions in ARs 1-4, the N-terminal 300 residues of wild type AnkA were modeled using Phyre 2 (Kelley et al., 2015). The top hit was 4RLV (RCSG Protein Data Bank) encoded as Ank1 (*Mus musculus*) and ANK2 (*Homo sapiens*). The crystal structure of 4RLV was determined to comprise 24 ANK repeats when complexed with its AnkR-specific auto-inhibitory segment (Wang et al., 2014). The alignment of 4RLV to the N-terminal 300 residues of AnkA identified with 287 residues (96% of AnkA N-terminal sequence) created a model with 100.0% confidence and 31% identity at a 3.49 Å resolution. The AnkA model aligned the first AnkA AR with AR2 of 4RVL, the second AnkA AR with AR5 of 4RVL, followed by a 499-residue gap and alignment of the third and fourth AnkA ARs with AR21 and AR22 of 4RVL, respectively (**Supplementary Figures 2**–**4**). The modeled AnkA structure demonstrated the specific locations of the 13^th^ residue mutations at the loops between α helices in predicted AnkA ARs. Impacts of mutations at these sites were also estimated to be negligible or absent using Missense3D and Phyre2 Run Investigator (**Supplementary Figure 4**). However, individual residue changes outside of the AR hydrophobic fold that centers on residue 13 are predicted to have potentially significant structural impacts (**Supplementary Table 2**).

### Single-Mutations of Two Consecutive N-Terminal ARs Reduces Nuclear Localization of AnkA

**Figure 3:**
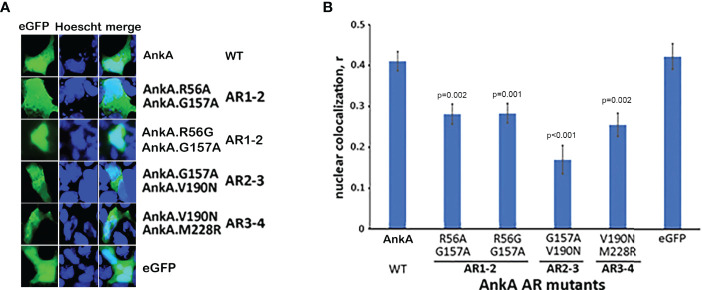
Single mutations in two adjacent N-terminal ARs significantly reduce nuclear localization of AnkA. HEK293T cells were transfected with eGFP plasmid constructs of AnkA or AnkA AR mutants in which two neighboring ankyrin repeats 1 & 2, 2 & 3 or 3 & 4 (AR1-2, AR2-3, AR3-4) had a single amino acid replacement. After fixing and washing, the cell nuclei were stained with Hoescht33342 and visualized with fluorescent microscopy. **(A)** Representative images of cells with eGFP-AnkA and dual-mutated ankyrin repeats are shown. **(B)** Nuclear colocalization of AnkA and mutants were determined by using the colocalization module of Olympus CellSense software. Data shown are mean ± SEM of Pearson correlation coefficient, r, from 20-40 ROIs containing 100-200 transfected cells of two independent experiments in duplicate. ANOVA *post hoc* Bonferroni corrected p values <0.05 relative to wild type AnkA are shown. Bar-20 µm.

In contrast to the inability of AnkA mutations in single ARs to inhibit nuclear localization, mutations in two consecutive ARs significantly decreased nuclear localization of mutant eGFP-AnkAs as demonstrated for AR mutation combinations AR1/AR2, AR2/AR3 and AR3/AR4 (**Figure 3**). While residue changes in AR1 and AR2 would not provide a dramatic reduction in hydrophobicity at the 13 position, and the changes in AR3 (AnkA.V190N) and AR4 (AnkA.M228R) would, these data demonstrate that pairs of adjacent N-terminal ARs could play a role in nuclear translocation of AnkA, similar to that seen in the eukaryotic RaDAR system (Lu et al., 2014).

### AnkA Is Transported by an Importin-β, RanGTP-Dependent Mechanism

**Figure 4:**
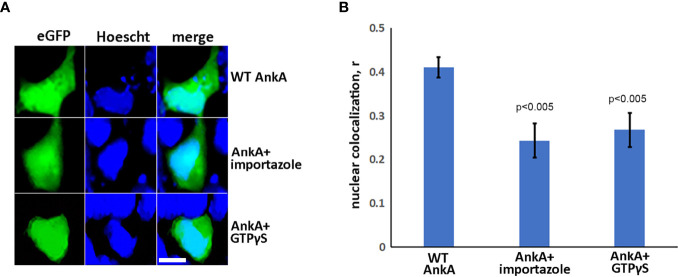
AnkA is transported into nucleus through importin-β, RanGTP-dependent nuclear-transporting system. HEK293T cells were transfected with eGFP-AnkA plasmid constructs and treated or sham-treated with 30 µM importazole or simultaneously loaded during transfection protocol with GTPγS, a non-hydrolysable analog of GTP. After 48 hours, cells were fixed, washed, stained with nuclear label Hoescht33342 and visualized by fluorescence microscopy. **(A)** Representative images of AnkA-transfected cells in the absence or presence of importazole or GTPγS. **(B)** Nuclear colocalization of AnkA proteins in the presence of importazole or GTPγS relative to untreated cells. Control cells transfected with pure eGFP plasmid construct are shown. The Pearson correlation coefficient, r, was used for the analysis. Data are mean ± SEM of 30-40 randomly selected ROIs containing 100-200 transfected cells of two independent experiments in duplicate. Student t-test p values compared to wild type AnkA are shown. Bar- 20µm.

**Figure 5:**
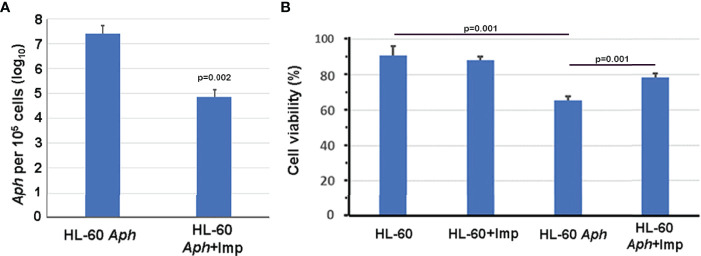
Importazole, an inhibitor of cargo binding to importin-β, inhibits *A. phagocytophilum* propagation in HL-60 cells **(A)** without inducing detrimental HL-60 cell viability **(B)**. *Aph*, A*. phagocytophilum*; Imp, importazole; ANOVA with Tukey *post hoc* p values <0.05 are shown. Error bars represent s.e.m.

**Figure 6:**
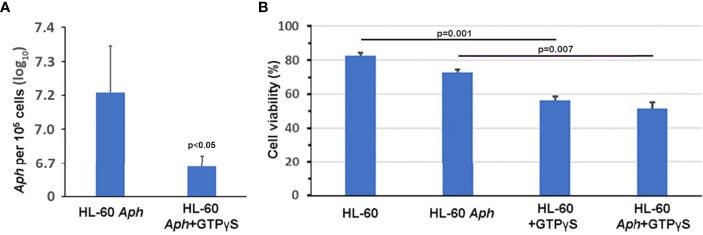
GTPγS, a non-hydrolysable GTP analog that inhibits RanGTP/RanGDP-dependent nuclear translocation by importin-α/β and RaDAR mechanisms and likely many other cellular processes, significantly impairs *A. phagocytophilum* growth *in vitro* **(A)** without further compromising viability of infected HL-60 cells **(B)**. *Aph*, A*. phagocytophilum*; Error bars represent s.e.m.

To investigate whether nuclear import of AnkA is mediated by the importin α/β pathway, we utilized importazole, a specific inhibitor of importin-β/RanGTP interaction (Soderholm et al., 2011), and GTPγS (guanosine 5’-O-[gamma-thio]triphosphate), a non-or slowly-hydrolysable GTP analog. We expected that the small molecule, cell-permeable importazole would interfere with importin-β/RanGDP transport across nuclear membrane, and its use should not alter nuclear localization if the RaDAR system was important. Additionally, we anticipated that loading cells with GTPγS would result in a rise of RanGTP concentration in the cytoplasm by suppressing activity of RanGTPase-activating protein (RanGAP). This would in turn block RanGTP/RanGDP shuttling across the nuclear membrane and thus import of AnkA - important in both importin α/β and RaDAR nuclear transport. We found that transfection of recombinant eGFP-AnkA into HEK293T cells that were separately loaded with importazole-β or GTPγS, resulted in significant reductions of AnkA nuclear localization (**Figure 4**). Moreover, when used with *A. phagocytophilum*-infected HL-60 cells, importazole inhibited bacterial propagation by >99% (p<0.005) without reducing cell viability (**Figure 5**). While GTPγS was partly toxic to HL-60 cells, the viability of GTPγS-treated HL-60 cells was not further diminished by *A. phagocytophilum* infection, whereas GTPγS inhibited *A. phagocytophilum* propagation by 71% (p<0.05) in treated HL-60 cells (**Figure 6**). The impact of the AnkA AR mutants on *A. phagocytophilum* growth could not be assessed since it is largely refractory to genetic manipulation.

### A Predicted Bipartite NLS in AR4

**Figure 7:**
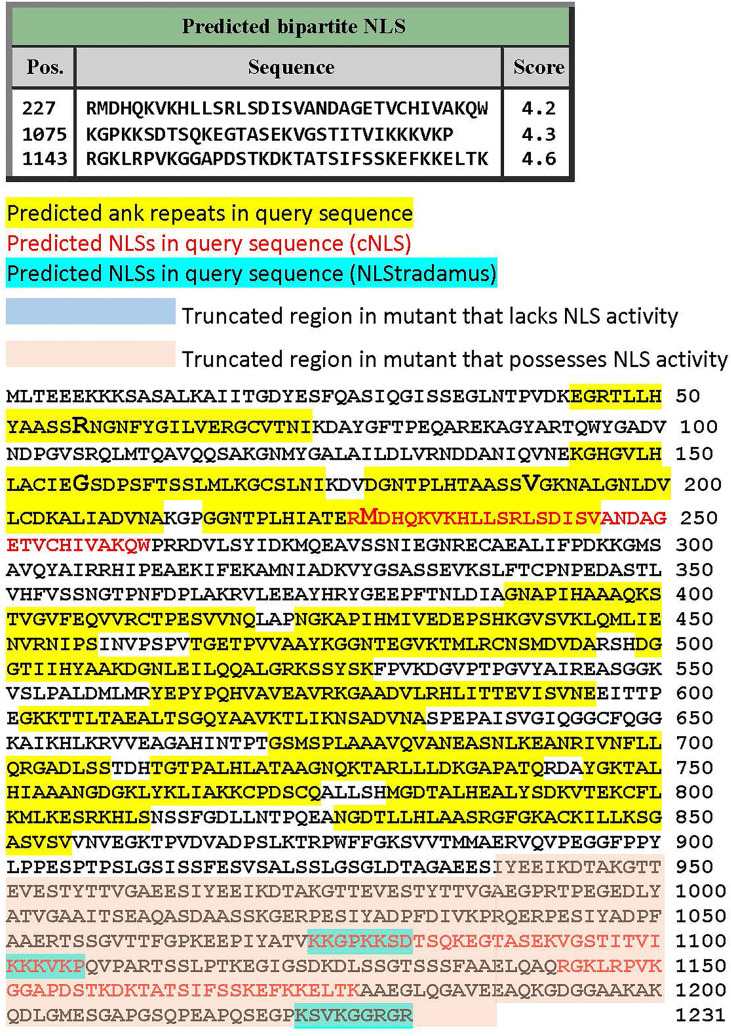
Prediction of importin-α/β pathway NLSs in full length *A. phagocytophilum* Webster^T^ strain AnkA. Three predictive algorithms were applied to identify putative NLSs. Shown is the full-length AnkA protein sequence with yellow highlights for the ankyrin repeats, ankyrin repeat mutations in enlarged font, NLS predicted by the cNLS Mapper algorithm (see text) in red text, and NLS predicted by NLStradamus (see text) in blue highlights. Note the predicted NLS overlapping the 4^th^ ankyrin repeat including the site of mutation that led to diminished nuclear translocation.

We previously used a range of publicly available NLS prediction algorithms to screen AnkA, and identified a putative bipartite NLS in the carboxy-terminus, that was disproven by creating truncation mutants (Rennoll-Bankert et al., 2015). Since dependency on the importin α/β system for AnkA nuclear localization and microbial propagation was shown, we applied additional alternative algorithms to discern the existence of previously unidentified NLSs. SeqNLS did not identify any putative NLSs, while NLStradamus identified putative NLSs only within the carboxy terminus that was already excluded as mechanistically important for AnkA nuclear import by experimental truncation of that region (Rennoll-Bankert et al., 2015). However, using cNLS Mapper (Kosugi et al., 2009b), three putative bipartite importin-β NLSs were identified, including two in the AnkA carboxy terminus (positions 1075-1106 and 1143-1177), and a single putative NLS at position 227-261 that overlaps AR4, including the AR4 mutation AnkA.M228R (**Figure 7**). cNLS Mapper predicted that the AnkA.M228R mutant sequence would not alter recognition of the NLS.

## Discussion

AnkA, a type IV secretion system effector protein of the tick-borne bacterium *A. phagocytophilum*, is essential for bacterial growth and has several roles in cellular infection and fitness (Lin et al., 2007; Sinclair et al., 2014; Rennoll-Bankert et al., 2015; O’Conor et al., 2021). The characteristic feature of AnkA is the presence of multiple ankyrin repeat motifs that span nearly 2/3 of the length of the protein (Scharf et al., 2011; Rennoll-Bankert et al., 2015). Depending on the strain, the number of ARs can vary from 8 to 15 (Scharf et al., 2011), but the N-terminal 4 ARs are highly conserved (Rennoll-Bankert et al., 2015). In general, ARs have multifunctional roles in protein-protein, protein-lipid and protein-DNA interactions. AnkA belongs to an increasingly large group of ARPs of prokaryotic origin with a very diverse functions (Al-Khodor et al., 2010; Islam et al., 2018). Understanding how the various protein domains, motifs, and structures influence functional properties informs approaches toward a better comprehension of pathogenesis. AnkA plays a critical role in modifying epigenetics of its host cell in mammals, the neutrophil (Garcia-Garcia et al., 2009b; Rennoll-Bankert et al., 2015; Sinclair et al., 2015; Dumler et al., 2016; Dumler et al., 2018). While AnkA appears to act as a nuclear chromatin organizer in the host cell *via* binding intergenic DNA locations to the nuclear lamina, its best known function is to bind DNA at host defense gene promoters (Garcia-Garcia et al., 2007; Garcia-Garcia et al., 2009b; Rennoll-Bankert et al., 2015; Dumler et al., 2016). It is at gene promoters that histone deacetylase-1 (HDAC1) is recruited after AnkA-DNA binding to deacetylate adjacent histone H3 and close chromatin accessibility to transcriptional activators and RNA polymerase. Additional nuclear functions for this prokaryotic protein are under study, including how it may affect the coordination of neutrophil transcriptional programs that enhance proinflammatory activities, delay apoptosis, and yet deactivate antimicrobial functions that enhance their fitness by promoting propagaton and transmission (Sinclair et al., 2014; Dumler et al., 2016; Dumler et al., 2018). Thus, understanding how AnkA enters the nucleus is a key to understanding disease pathogenesis.

ARs located in the middle or carboxy-end of AnkA play a role in binding to host DNA and in promoter silencing, presumably through recruitment of HDAC1 (Rennoll-Bankert et al., 2015). At the same time, truncation of the N-terminus of AnkA that includes the first four ARs results in a significant decrease of nuclear localization of the protein, implying the presence of a previously undetected nuclear localization sequence in this region. Classical importin-dependent nuclear localization requires bipartite or monopartite cargo NLS interactions with ARM motifs in importin-α before binding importin-β and transiting the nuclear pore complex, where RanGTP binding releases the cargo protein (Lu et al., 2021). A critical component of this process relies on cytoplasmic RanGDP complexed with nuclear transport factor 2 (NTF2) to enter the nucleus before conversion to RanGTP (Ribbeck et al., 1998).

In contrast, structural analysis of some of human ARPs suggest that alternate NLS interactions take place in a hydrophobic pocket formed by at-least two ARs of the same ARP or with a tandem of ARs of two ARPs (Lu et al., 2014). For a group of ARPs that are imported into the nucleus, this transport requires the RaDAR pathway that requires two adjacent ARs, importin-β-independence, and involvement of RanGDP binding to translocate into the nucleus. The distinguishing feature of these ARPs is that their nuclear transport is defined by ARs with a hydrophobic residue at the 13^th^ position that mediates binding to similar hydrophobic residues in RanGDP; replacement of these with hydrophilic residues (e.g. K, R, E, Q) in two consecutive ARs abrogates nuclear import of the ARPs by the RaDAR system (Lu et al., 2014). While the tertiary structure of AnkA is not yet fully resolved, truncation of the N-terminal ARs leads to loss of nuclear importation that is restored when the SV40 NLS is added to the C-terminus. This also restores typical DNA binding of AnkA, thus focusing attention on the unique NLS properties of the N-terminal ARs and implying that AnkA might also be transported by the RaDAR system (Lu et al., 2014; Rennoll-Bankert et al., 2015).

Here, we interrogated the potential involvement of the RaDAR nuclear localization mechanism and found that point mutations of two consecutive ARs, such as AR1/AR2, AR2/AR3 or AR3/AR4 significantly reduced nuclear importation of AnkA. Although consistent with the RaDAR mechanism, single mutations at ARs 1-4 did not reduce AnkA nuclear localization. There is controversy regarding the requirement for the two consecutive AR principle of RaDAR function; for example, Ank13 of *O. tsutsugamushi* uses the RaDAR mechanism, but mutation of a single AR 13^th^ residue is sufficient to reduce nuclear entry (Adcox et al., 2021). While replacement of hydrophobic with hydrophilic 13^th^ residues of the consecutive AR3/AR4 resulted in a reduction of nuclear localization, mutations that increase hydrophobicity at AR1/AR2 and AR2/AR3 also reduce nuclear localization, a finding inconsistent with a stringent interpretation of the RaDAR mechanism. Mutations at the 13^th^ residues of ARs do not lead to structural changes in the RanGDP binding site (Lu et al., 2014), and such changes are not predicted to disrupt key structural features of the protein (**Supplementary Figure 2**), yet these and our supplemental findings (**Supplementary Table 1**) suggest otherwise. While individual deletions of AR1, 2, 3 and 4 could be informative, the likely disruption to protein structure could confound interpretation of their nuclear localization (Lu et al., 2014). Moreover, our data with mutations not relevant to RaDAR nuclear importation that affect the same ARs also suggests the importance of AR structural integrity for AnkA nuclear transport.

In contrast, the use of importazole, a specific inhibitor of importin-β-dependent cargo protein nuclear delivery, showed that AnkA transport was importin-α/β-dependent. The identification of importin-dependence and the inhibition of *A. phagocytophilum* growth in its presence prompted a further search for an alternate NLS not previously identified. While prediction algorithms for NLSs vary, each possesses weaknesses; for greatest validity the approach would be confirmed by several methods and experimentally (Lin and Hu, 2013). The predicted colocalization of a putative bipartite importin-α NLS that overlaps AR4 perhaps offers support for the former hypothesis, although the M228R mutation is not predicted to significantly modify structure or classical bipartite NLS function; definitive evidence will require additional mutational studies. These data suggest that the RaDAR mechanism is either a supplemental, redundant or minor contributor for nuclear localization activity of AnkA. This is reasoned because if the RaDAR system, that by definition does not require importin, was critical for the essential protein AnkA nuclear localization, inhibition of importin-dependent nuclear translocation would not have the profound effect on *A. phagocytophilum* propagation noted here. While it appears that AnkA utilizes traditional nuclear import to enter the nucleus, the supplemental use of the RaDAR mechanism might provide an evolutionary advantage in pathogen fitness, although propagation dependence on importin contradicts that hypothesis. What is less clear is the impact of amino acid residue substitutions in proximity to position 13, where Lu et al. found AR structural stability at positions 3, 5, 13 and 14 (Lu et al., 2014). These findings suggest instability at other residues in ARs or to non-AR amino acid sequence could impact protein structure and have profound effects on protein function, further supporting the primacy of the importin-α/β nuclear import for AnkA.

Similar to *A. phagocytophilum* AnkA, *Orientia tsutsugamushi* ARPs Ank1 and Ank6 require importin-β, but Ank13 utilizes the RaDAR pathway (Evans et al., 2018; Adcox et al., 2021), suggesting the coexistence of both processes utilized by a single bacterium, albeit for distinct proteins, and that perhaps two consecutive ARs with 13^th^ position hydrophobic residues are not required for prokaryotic proteins. Interestingly, a related tick-borne pathogen, *Ehrlichia chaffeensis*, uses an AR-rich protein, p200, to modify transcriptional activity of its host monocyte (Zhu et al., 2009). The p200 protein contains about 20 ARs among its 1422 residues tightly clustered in 3 locations. However, as in the case with *O. tsutsugamushi* Ank1 and Ank6, an analysis of amino acid sequences of p200 ARs reported in Protein databases and GenBank do not meet stringent criteria that define the RaDAR nuclear import pathway. Given the increasing ubiquity of ARPs discovered in prokaryotes through genome analyses, future studies should address whether they use the RaDAR transporting system as do many human ARPs, whether they use classical importin-α/β-dependent nuclear transport pathway, serve supplemental or redundant roles, or do not contribute to nuclear importation. Since different dual combinations of N-terminal AR mutations affect AnkA nuclear localization, future studies of AnkA tertiary structure will be of critical importance to understand a role for the four N-terminal ARs in nuclear import or other AnkA functions.

Given the essential nature and importance noted for AnkA in the fitness of *A. phagocytophilum*, understanding the function of all protein motifs and domains is essential to understanding pathogenicity and to ultimately develop improved strategies for disease control. Additionally, the application of ankyrin repeats for use as biological tools and pharmaceuticals (Boersma, 2018; Mittl et al., 2020) suggests that improved properties and ranges of function could be discerned by studying those features already selected for specific properties by microbes, like *A. phagocytophilum*.

## Experimental Procedures

### Cell Culture

HEK293T, human embryonic kidney cells (CRL3216, ATCC), were grown in RPMI 1640 (GIBCO) medium supplemented with 10% FBS (GemCell, Gemini), 2 mM L-glutamine and 1 mM sodium pyruvate at 37°C and 5% CO_2_. *A. phagocytophilum* (*Aph*) Webster^T^ strain was maintained in HL-60 cells, a human promyelocytic cell line, that were cultured in RPMI 1640 with 10% FBS supplemented with 2 mM glutamine and 1 mM pyruvate.

### Construction of Point Mutation AnkA Plasmids

To preclude major structural variations of AnkA with AR deletions that would potentially impact its binding, we avoided deletion of individual N-terminal ARs in favor of specific point mutations that would better elucidate potential RaDAR nuclear transport involvement. Plasmids pEGFPC1-AnkA and N-terminal ankyrin repeat mutants of AnkA were generated (GenScript; Piscataway, NJ) according to the full AnkA sequence of *A. phagocytophilum* Webster^T^ strain (WP_020849331.1). We modified the 13^th^ amino acid residues in each of the first four N-terminal ARs to hydrophilic or neutral residues anticipating a change in hydrophobic interactions with RanGDP that would impact nuclear localization (**Figure 1**). In total, 11 single or double AR mutations of AnkA were created. AR1 and AR2 of AnkA differ from human ARs involved in RaDAR nuclear transport in that they have a hydrophilic and aliphatic neutral residue at the 13th position, arginine and glycine, respectively. Thus, recombinant mutants of the protein were created in which the 13th residues were replaced with more hydrophobic or neutral/hydrophilic residues, alanine (AnkA.R56A) or glycine (AnkA.R56G) for AR1, and alanine (AnkA.G157A) or arginine (AnkA.G157R) for AR2. In contrast, AR3 and AR4 of AnkA have amino acid sequences identical to ARs of many human proteins involved in RaDAR nuclear transport, such as the presence of a highly conserved LH pair at the 5th and 6th positions and of hydrophobic residues at the 13th position, valine and methionine, respectively. We replaced these with hydrophilic asparagine (AnkA.V190N) because of its similar molecular size, and arginine (AnkA.M228R) because of its similar size and structure, respectively. Wild type eGFP-AnkA and mutated plasmids were transfected into HEK293T cells and their nuclear localizations were compared.

### Cell Transfection

For cell transfection and subsequent visualization, HEK293 cells were seeded into collagen-coated 8-chamber optical glass slides (LabTek) at 4-5x10^4^ cells/per well in 400 µL of growth medium. After 24h, the attached cells were transfected with the AnkA or AnkA N-terminal ankyrin repeat mutant plasmids. Transfection (FuGENE HD Promega) was achieved using 2 µg of plasmid DNA and 3:1 ratio of the reagent (6 µL) per well, in accordance with manufacturer recommendations. After 48h incubation, cells were washed in PBS, fixed for 10 min in 4% paraformaldehyde, washed in PBS and then stained with Hoescht 33342, a cell-permeable nuclear label, at 1:1000 dilution for 3 minutes. After final washing in PBS, the chamber walls were removed and the dried optical slides were fixed, and labeled cells were mounted (ProLong Diamond Antifade mounting solution, Invitrogen) and visualized using fluorescence microscopy.

### Image Analysis

Slides were visualized using an Olympus BX-40 fluorescent microscope equipped with DP74 camera, 40x-objective and precision stage (ProScanIII from Prior). For image analysis, the Olympus CellSense Dimension software with the colocalization software module was used (Casavan et al., 2021). In this program, Pearson’s correlation coefficient accounts only for the similarity of shapes between two colocalized images highlighted by distinct fluorescence in the nucleus, and does not depend upon image pixel intensity values. 20-40 regions of interest (ROIs) containing 100-200 transfected nuclei stained with Hoescht (blue) were randomly selected and examined for colocalization with eGFP fluorescence (**Supplementary Figure 1** shows an example of this analysis). The images used include eGFP fluorescent signal within, above and below the nucleus, such that the minimal colocalization signal will never be zero, but that with increased nuclear eGFP presence, will increase. To optimize visual differences in colocalized signal, for each image displayed, we utilized the image/adjustment/levels feature in Photoshop 22 to remove red (output level 0), and adjusted both green and blue channels. Based on this colocalization analysis and comparing wild type to mutants, Pearson coefficients, R(r), from two independent experiments done in duplicate, were statistically analyzed using either Student’s t-test for comparing two conditions or one-way ANOVA with *post-hoc* Tukey test for multiple conditions, or for comparisons related to control only, Bonferroni corrections were performed.

### Bacterial Growth and Infectivity

To evaluate the infectivity of *A. phagocytophilum* in HL-60 cells we used a cytological Romanowsky staining method; trypan blue exclusion was used with the Countess II (Invitrogen) automatic cell counter to determine cell viability. To study the role of importin-dependent nuclear transport in *A. phagocytophilum* growth, 3x10^5^ HL-60 cells were seeded in 12-well plates (Costar) containing 1 mL of growth medium in the presence or absence of 10 µM importazole, a specific nuclear transport inhibitor (Soderholm et al., 2011). To investigate the role of RanGTP/RanGDP-dependent nuclear transport system in *A. phagocytophilum* growth, 3x10^5^ HL-60 cells were loaded with non-hydrolysable GTP analog, GTPγS using the transfection protocol described for cell transfection with AnkA plasmids. Here, nanoparticles were incubated with 100 µM GTPγS and then added to *A. phagocytophilum*-infected or non-infected HL-60 cells. To ascertain the effects of importazole and GTPγS on *A. phagocytophilum* propagation, 10^5^ infected HL-60 cells were added to 9 x 10^5^ uninfected HL-60 cells to achieve 10% infected cells, for an average of 1 bacterium per cell. These and 10^6^ control uninfected HL-60 cells, both in 5 mL, were supplemented to achieve final concentrations of 10 µM importazole or 100 µM GTPγS. After 3 days of incubation, control treated and untreated cells and *A. phagocytophilum*-infected, importazole/GTPyS-treated and sham-treated HL-60 cells were collected for cell count, viability evaluation, and for quantitation of *A. phagocytophilum* by qPCR targeting *A. phagocytophilum msp2* (Reller and Dumler, 2020). This qPCR assay is based on a 5-prime nuclease assay targeting a highly conserved region of the multicopy *msp2*, for which at least 100 copies exist in the *A. phagocytophilum* genome. The assay has been demonstrated to detect up to 86 distinct copies in the *A. phagocytophilum* Webster^T^ strain genome. Thus, using *msp2* plasmid standards ranging from 10^1^ to 10^6^ copies was used to determine the *msp2* copies per µL in technical duplicates or triplicates for each condition with 3 biological replicates. DNA was prepared from 10^5^ infected or uninfected, treated or untreated cells suspended in 200 µL, and wells of a 384 well PCR plate received 1-3 µL of DNA in addition to master mix, in a final 10 µL well volume. To establish the number of bacteria, the number of *msp2* copies/µL in each condition was divided by the volume tested and by 86 copies/*A. phagocytophilum* genome to obtain *A. phagocytophilum*/µL, then multiplied by 200 to obtain the total number of bacteria in 10^5^ cells.

### Bioinformatic Screening of β-Importin Nuclear Localization Signals in AnkA

While AnkA had initially been screened for the presence of putative NLSs (Rennoll-Bankert et al., 2015), the dependence on importazole and GTP hydrolysis for translocation prompted an additional search using recently developed publically-available software programs that utilize distinct search algorithms, including SeqNLS (http://mleg.cse.sc.edu/seqNLS/, using Final-score cutoffs of 0.3, 0.5 and default 0.86) (Lin and Hu, 2013); NLStradamus (https://predictprotein.org/, using 2 state HMM static and dynamic, and 4 state HMM static models with Viterbi and Posterior prediction models [0.4 prediction cutoff]) (Nguyen Ba et al., 2009); NLSdb (https://rostlab.org/services/nlsdb/) (Nair et al., 2003); and NLS Mapper cNLS (https://nls-mapper.iab.keio.ac.jp/cgi-bin/NLS_Mapper_form.cgi, using cut-off scores of 5.0, 4.0, and 3.0, Entire region) (Kosugi et al., 2009a; Kosugi et al., 2009b). Predicted NLSs were mapped against the AnkA protein sequence with and without mutated residues (**Figure 7**).

### AnkA N-Terminal Ankyrin Repeat Structural Modeling

To predict impact of point mutations in the N-terminal region of AnkA, we modeled the first 300 amino acids to the top ranked model in a Phyre2 analysis. The model pdb file was then used to interrogate single mutants constructed for the *in vitro* studies using Missense 3D which allowed prediction of each potential residue substitution toward stability of protein structure and visualization of mutant residues on the top model’s structure (Kelley et al., 2015; Ittisoponpisan et al., 2019). Additional analysis of the AnkA N-terminal 300 residue model was analyzed for the effects of structural changes with single residue alterations at every position using SuSPect (Yates et al., 2014).

### Statistical Analysis

Two-tailed Student t-tests were performed by using Excel software (Microsoft, Redmond, WA). For multiple comparisons, one-way ANOVA with *post-hoc* Tukey HSD tests were performed, or for comparisons related to control only, Bonferroni corrections were performed (https://astatsa.com/OneWay_Anova_with_TukeyHSD/). Values of p<0.05 were considered significant.

## Data Availability Statement

The original contributions presented in the study are included in the article/**Supplementary Material**. Further inquiries can be directed to the corresponding author.

## Author Contributions

JD, YK and JW conceived and designed the study. YK, JW, and EC performed the experiments. YK and JD performed the statistical analysis. YK and JD wrote the paper. JW, EC, and DG participated in the drafting of the manuscript. All authors contributed to the preparation of the article and approved the submitted version.

## Funding

This work was supported by grant R01-AI044102 from the National Institutes of Allergy and Infectious Diseases/National Institutes of Health to JD, and by grant PAT-74-3977 from the Uniformed Services University of the Health Sciences to JD.

## Conflict of Interest

The authors declare that the research was conducted in the absence of any commercial or financial relationships that could be construed as a potential conflict of interest.

## Publisher’s Note

The opinions expressed herein are those of the author(s) and are not necessarily representative of those of the Uniformed Services University of the Health Sciences (USUHS), the Department of Defense (DOD); or, the United States Army, Navy, or Air Force.

## Publisher’s Note

All claims expressed in this article are solely those of the authors and do not necessarily represent those of their affiliated organizations, or those of the publisher, the editors and the reviewers. Any product that may be evaluated in this article, or claim that may be made by its manufacturer, is not guaranteed or endorsed by the publisher.

## Supplementary Material

Supplementary Figure 1 Example of Fluorophore Colocalization analysis. A 2D field identified among HEK cells transfected with pEGFPC1-ankA (wild type) and imaged to visualize transfected cells (green) and all cell nuclei (blue) demonstrate a modest transfection efficiency (top panel). Representative fields that included eGFPexpression were selected for imaging. Among cells that display transfection, approximately 25 in the field shown here, colocalization of transfected shape by pixels was identified by designating 100-200 regions of interest (ROI) (bottom panel and insert). From these, the Pearson’s correlation coefficient (r) was calculated to estimate the degree of overlap of the two signals. Values of r vary between -1 and +1, with clear colocalization indicated by positive r values closer to 1.

Supplementary Figure 2 The AnkA N-terminal 300 residue structure was modeled by alignment using Phyre2 to identify the best match, 4RLV (ANK2). Using this model, the location and impact of changes in individual residues was assessed using Missense3D. The specific insertions are shown on the c4RLV models of AnkA ARs 1-4 by AnkA AR, and labeled with the specific residue change. Note that the model c4RLV model structure results in two non-contiguous templates resulting from a large gap in 4RLV compared to AnkA. AR1 and 2 are on one template; AR3 and 4 on a different template. No residue change was predicted to cause any structural damage.

Supplementary Figure 3 Alignment of AnkA modeled by Phyre2 on 4RLV. ARs in AnkA are shown in yellow highlights, those in 4RLV are shown in green. Dashes indicate gaps in alignment. Note that AnkA AR2 spans a large gap in 4RLV. AnkA ARs 1-4 correspond to ARs 2, 5/20, 21, and 22 in 4RLV.

Supplementary Figure 4 Detailed alignment of AnkA N-terminal 300 residues by Phyre2 to 4RLV showing predicted secondary structure for both AnkA and 4RLV templates. Yellow highlights show AnkA ARs.
