# Supplementary figures and images for: Anaplasma phagocytophilum Ankyrin A Protein (AnkA) Enters the Nucleus Using an Importin-β-, RanGTP-Dependent Mechanism
Source: Front Cell Infect Microbiol. 2022 May 26;12:828605. doi: 10.3389/fcimb.2022.828605 (PMC9204287; doi:10.3389/fcimb.2022.828605)

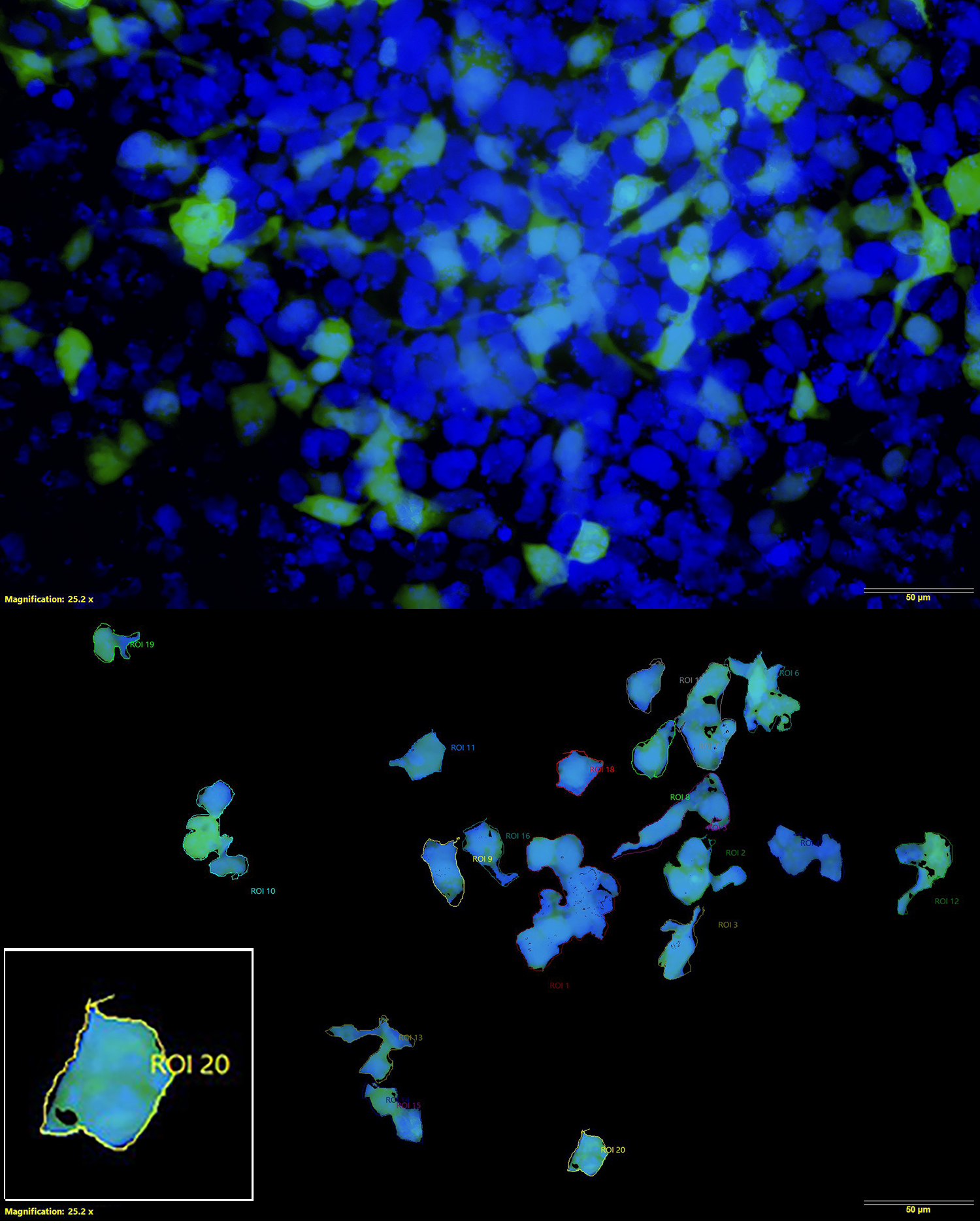

Supplement: Supplementary Figure 1 — Example of Fluorophore Colocalization analysis. A 2D field identified among HEK cells transfected with pEGFPC1-ankA (wild type) and imaged to visualize transfected cells (green) and all cell nuclei (blue) demonstrate a modest transfection efficiency (top panel). Representative fields that included eGFPexpression were selected for imaging. Among cells that display transfection, approximately 25 in the field shown here, colocalization of transfected shape by pixels was identified by designating 100-200 regions of interest (ROI) (bottom panel and insert). From these, the Pearson’s correlation coefficient (r) was calculated to estimate the degree of overlap of the two signals. Values of r vary between -1 and +1, with clear colocalization indicated by positive r values closer to 1. [file Image_1.jpeg]

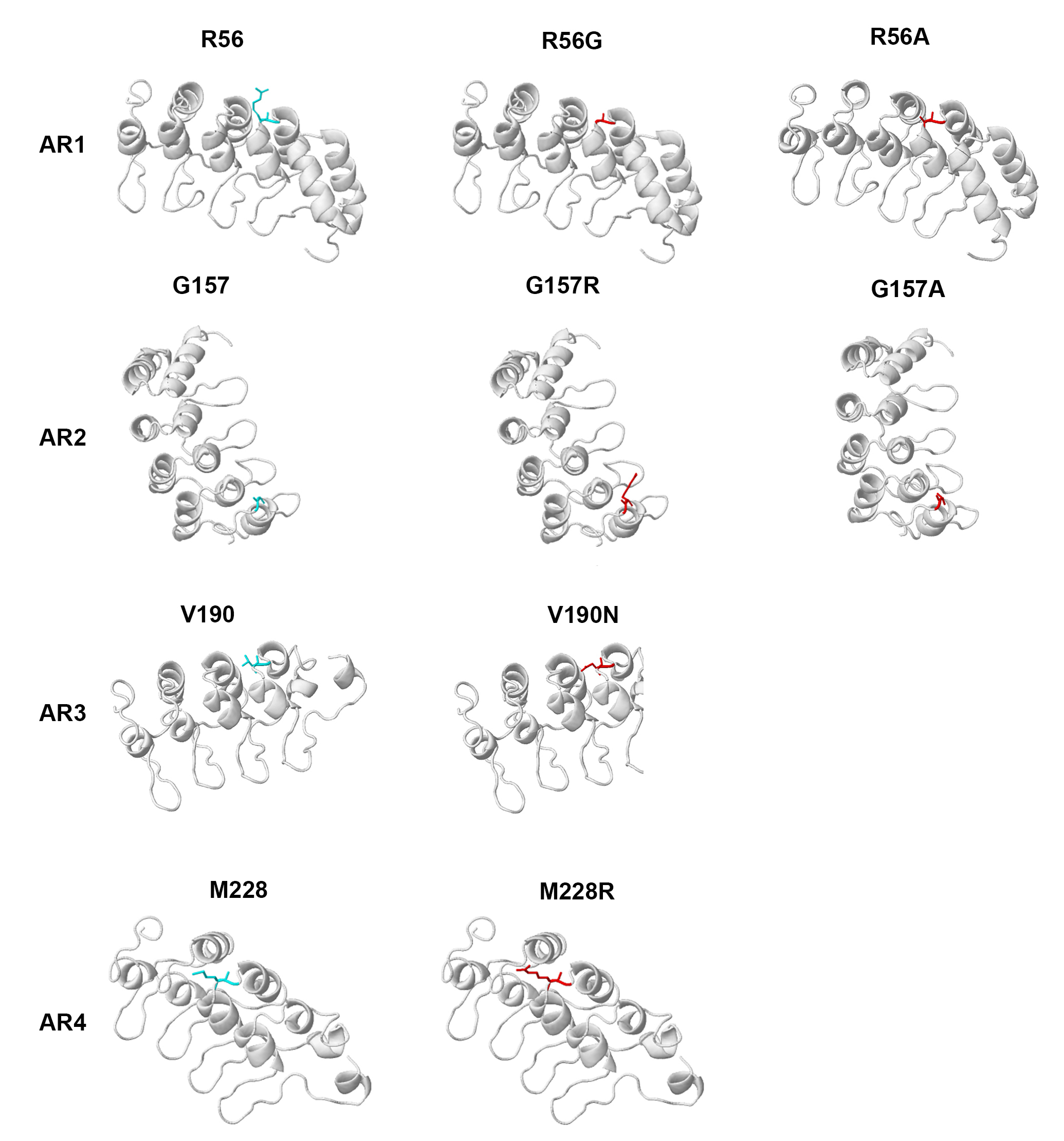

Supplement: Supplementary Figure 2 — The AnkA N-terminal 300 residue structure was modeled by alignment using Phyre2 to identify the best match, 4RLV (ANK2). Using this model, the location and impact of changes in individual residues was assessed using Missense3D. The specific insertions are shown on the c4RLV models of AnkA ARs 1-4 by AnkA AR, and labeled with the specific residue change. Note that the model c4RLV model structure results in two non-contiguous templates resulting from a large gap in 4RLV compared to AnkA. AR1 and 2 are on one template; AR3 and 4 on a different template. No residue change was predicted to cause any structural damage. [file Image_2.jpeg]

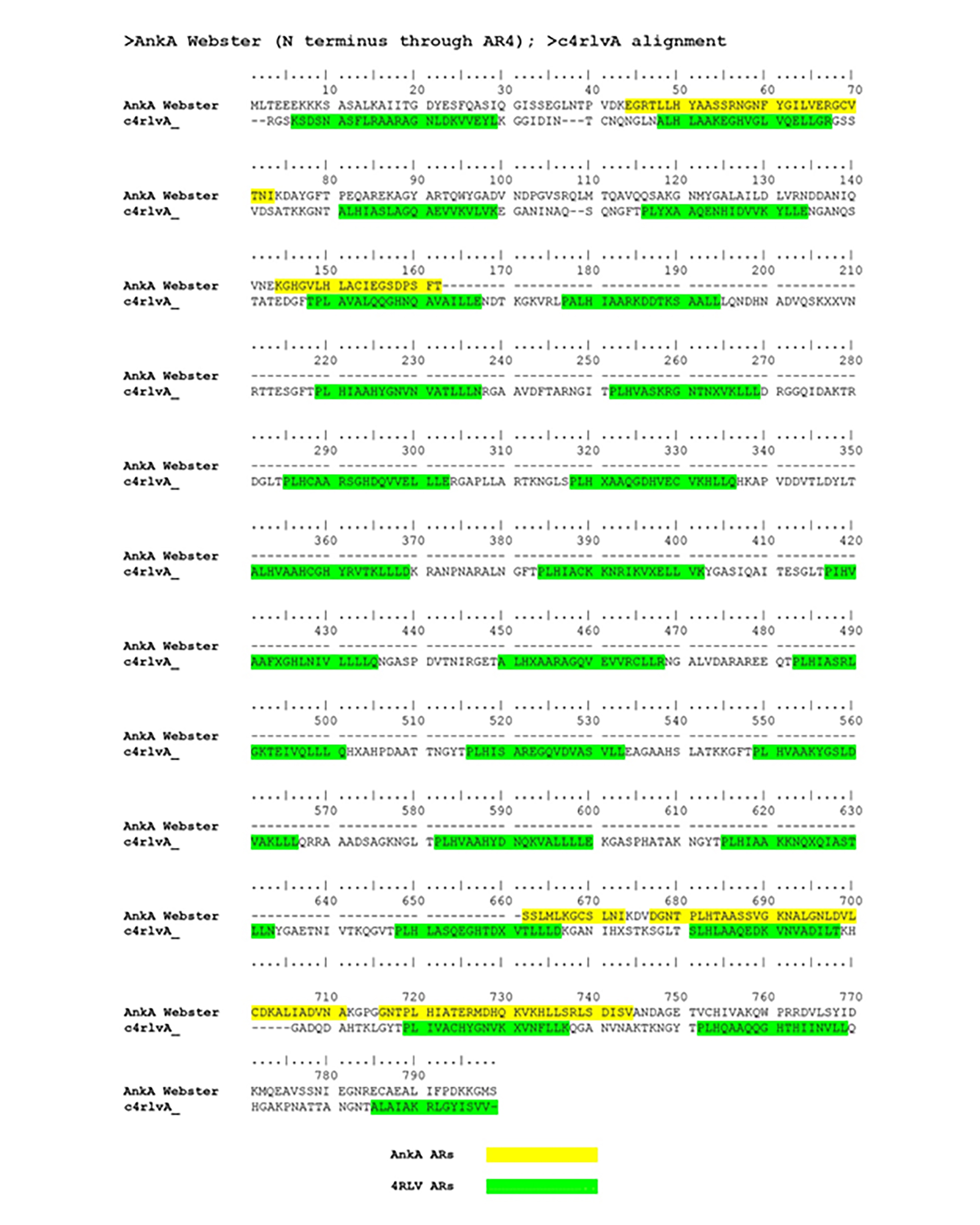

Supplement: Supplementary Figure 3 — Alignment of AnkA modeled by Phyre2 on 4RLV. ARs in AnkA are shown in yellow highlights, those in 4RLV are shown in green. Dashes indicate gaps in alignment. Note that AnkA AR2 spans a large gap in 4RLV. AnkA ARs 1-4 correspond to ARs 2, 5/20, 21, and 22 in 4RLV. [file Image_3.jpeg]
