# Supplementary material for: Anaplasma phagocytophilum Ankyrin A Protein (AnkA) Enters the Nucleus Using an Importin-β-, RanGTP-Dependent Mechanism
Source: Front Cell Infect Microbiol. 2022 May 26;12:828605. doi: 10.3389/fcimb.2022.828605 (PMC9204287; doi:10.3389/fcimb.2022.828605)
Supplement: Supplementary Figure 4 — Detailed alignment of AnkA N-terminal 300 residues by Phyre2 to 4RLV showing predicted secondary structure for both AnkA and 4RLV templates. Yellow highlights show AnkA ARs. [file Image_4.pdf]

Return to main results Retrieve Phyre Job Id Fetch

Job Description	Undefined		
Confidence	100.00%	Date	Tue Mar 15 21:02:29 GMT 2022
Rank	1	Aligned Residues	287
% Identity	31%	Template	c4rlvA_
PDB info	**PDB header:**structural protein	**Chain:** A: **PDB Molecule:**ankyrin-1, ankyrin-2;	**PDBTitle:** crystal structure of ankb 24 ankyrin repeats in complex with ankr2 autoinhibition segment **PDB Entry:** PDBe RCSB PDBj
Resolution	3.49 Å		
Model Dimensions (Å)	**X**:119.265 **Y**:73.766 **Z**:102.875		

Show / Hide SS confidence

Show / Hide Conservation and Alignment quality

Insertion relative to template

Deletion relative to template

Catalytic residue from the CSA

Detailed help on interpreting your alignment

3 . . . . . . 10 . . . . 20 . . . . 30 . . . . 40 . . . . 50 . . . . 60 . .

Predicted Secondary structure

Query Sequence T E E E K K K S A S A L K A I I T G D Y E S F Q A S I Q G I S S E G L N T P V D K E G R T L L H Y A A S S R N G N F Y G

Template Sequence R G S K S D S N A S F L R A A R A G N L D K V V E Y L K G G I D I N . . . T C N Q N G L N A L H L A A K E G H V G L V Q

Template Known Secondary structure T T T T T T T . . . T T T

Template Predicted Secondary structure . . .

1630 . . . . 1640 . . . . 1650 . . . . 1660 . . . . 1670 . . . . 1680 . . . . . .

```
                                  63      70        80        90        100       110       120
Predicted Secondary structure
Query Sequence                    ILVERGCVTNIKDAYGFTPEQAREKAGYARTQWYGADVNDPGVSRQLMTQAVQQSAKGNM
Template Sequence                 ELLGRGSSVDSATKKGNTALHIASLAGQAEVVKVLVKEGANINAQ..SQNGFTPLYXAAQ
Template Known Secondary structure    TT  TT  TT        T         TT  TT ..TT
Template Predicted Secondary
structure                                                                  ..
                                  2082    2090      2100      2110      2120      2130

                                  123     130       140       150       160
Predicted Secondary structure                                                  ....................
Query Sequence                    YGALAILDLVRNDDANIQVNEKGHGVLHLACIEGSDPSFT....................
Template Sequence                 ENHIDVVKYLLENGANQSTATEDGFTPLAVALQQGHNQAVAILLENDTKGKVRLPALHIA
Template Known Secondary structure TT         TT  TT    TTS         TT
Template Predicted Secondary
structure
                                  2140      2150      2160      2170      2180      2190

Predicted Secondary structure     ............................................................
Query Sequence                    ............................................................
Template Sequence                 ARKDDTKSAALLQNDHNADVQSKXXVNRTTESGFTPLHIAAHYGNVNVATLLLNRGAAV
Template Known Secondary structure    T        TSS     S GGGSGGG   TTS          T            TT  T
Template Predicted Secondary
structure
                                  2200      2210      2220      2230      2240      2250

Predicted Secondary structure     ............................................................
Query Sequence                    ............................................................
Template Sequence                 DFTARNGITPLHVASKRGNTNXVKLLLDRGGQIDAKTRDGLTPLHCAARSGHDQVVELLL
Template Known Secondary structure T  BTTTB          T          TT  TT   TT          T
Template Predicted Secondary
structure
                                  2260      2270      2280      2290      2300      2310

Predicted Secondary structure     ............................................................
Query Sequence                    ............................................................
Template Sequence                 ERGAPLLARTKNGLSPLHXAAQGDHVECVKHLLQHKAPVDDVTLDYLTALHVAAHCGHYR
Template Known Secondary structure  TT  TT   TT        TT           TT  TT   TT           TT
Template Predicted Secondary
structure
                                  2320      2330      2340      2350      2360      2370

Predicted Secondary structure     ............................................................
Query Sequence                    ............................................................
Template Sequence                 VTKLLLDKRANPNARALNGFTPLHIACKKNRIKVXELLVKYGASIQAITESGLTPIHVAA
```

Template Known Secondary structure: T T T T T T S T T T T T T T T
Template Predicted Secondary structure
2380 . . . . 2390 . . . . 2400 . . . . 2410 . . . . 2420 . . . . 2430 . . . .

Predicted Secondary structure: . . . .
Query Sequence: . . . .
Template Sequence: F X G H L N I V L L L Q N G A S P D V T N I R G E T A L H X A A R A G Q V E V V R C L L R N G A L V D A R A R E E Q T
Template Known Secondary structure: T T T T T T T T T T T T T G G G
Template Predicted Secondary structure
2440 . . . . 2450 . . . . 2460 . . . . 2470 . . . . 2480 . . . . 2490 . . . .

Predicted Secondary structure: . . . .
Query Sequence: . . . .
Template Sequence: P L H I A S R L G K T E I V Q L L L Q H X A H P D A A T T N G Y T P L H I S A R E G Q V D V A S V L L E A G A A H S L A
Template Known Secondary structure: T T T T T T T T T T T T T T
Template Predicted Secondary structure
2500 . . . . 2510 . . . . 2520 . . . . 2530 . . . . 2540 . . . . 2550 . . . .

Predicted Secondary structure: . . . .
Query Sequence: . . . .
Template Sequence: T K K G F T P L H V A A K Y G S L D V A K L L L Q R R A A A D S A G K N G L T P L H V A A H Y D N Q K V A L L L L E K G
Template Known Secondary structure: T T T T T T T T T T T T T T T
Template Predicted Secondary structure
2560 . . . . 2570 . . . . 2580 . . . . 2590 . . . . 2600 . . . . 2610 . . . .

163
Predicted Secondary structure: . . . .
Query Sequence: . . . . S
Template Sequence: A S P H A T A K N G Y T P L H I A A K K N Q X Q I A S T L L N Y G A E T N I V T K Q G V T P L H L A S Q E G H T D X V T
Template Known Secondary structure: T T S S T T T T S S T T T T
Template Predicted Secondary structure
2620 . . . . 2630 . . . . 2640 . . . . 2650 . . . . 2660 . . . . 2670 . . . .

164 . . . . . 170 . . . . 180 . . . . 190 . . . . 200 . . . . 210 . . . . 220 . . .
Predicted Secondary structure
Query Sequence: S L M L K G C S L N I K D V D G N T P L H T A A S S V G K N A L G N L D V L C D K A L I A D V N A K G P G G N T P L H I
Template Sequence: L L L D K G A N I H X S T K S G L T S L H L A A Q E D K V N V A D I L T K H . . . . . G A D Q D A H T K L G Y T P L I V
Template Known Secondary structure: T T T S S S T T T T T . . . . . T S S S S
Template Predicted Secondary structure: . . . . . .
2680 . . . . 2690 . . . . 2700 . . . . 2710 . . . . 2720 . . . . 2730 . . . .

224 . . . . . 230 . . . . 240 . . . . 250 . . . . 260 . . . . 270 . . . . 280 . . .

Predicted Secondary structure
Query Sequence A T E R M D H Q K V K H L L S R L S D I S V A N D A G E T V C H I V A K Q W P R R D V L S Y I D K M Q E A V S S N I E G
Template Sequence A C H Y G N V K X V N F L L K Q G A N V N A K T K N G Y T P L H Q A A Q Q G H T H I I N V L L Q H G A K P N A T T A N G
Template Known Secondary structure T T T T T T T S S S T T T T T T T T T S S S
Template Predicted Secondary structure

2735 . . . . 2740 . . . . 2750 . . . . 2760 . . . . 2770 . . . . 2780 . . . . 2790 . . . .

284 . . . . . 290 . . . .

Predicted Secondary structure
Query Sequence N R E C A E A L I F P D K K G M
Template Sequence N T A L A I A K R L G Y I S V V
Template Known Secondary structure T T T T T T T T T
Template Predicted Secondary structure

2795 . . . . 2800 2810

Download: Text version FASTA pairwise alignment 3D Model in PDB format

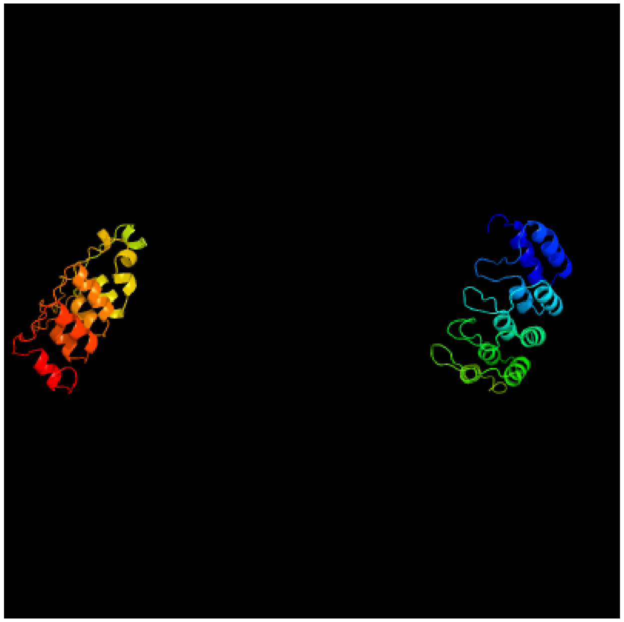

View in JSmol

Send structure to FirstGlance for more viewing options

Phyre is now FREE for commercial users!

All images and data generated by Phyre2 are free to use in any publication with acknowledgement

Accessibility Statement

Please cite: The Phyre2 web portal for protein modeling, prediction and analysis
Kelley LA *et al. Nature Protocols* 10, 845-858 (2015) [paper] [Citation link]

© Structural Bioinformatics Group, Imperial College, London
Lawrence Kelley, Michael Sternberg
Disclaimer
Terms and Conditions

Phyre2 is part of **Genome3D**
