# Supplementary material for: Anaplasma phagocytophilum Ankyrin A Protein (AnkA) Enters the Nucleus Using an Importin-β-, RanGTP-Dependent Mechanism
Source: Front Cell Infect Microbiol. 2022 May 26;12:828605. doi: 10.3389/fcimb.2022.828605 (PMC9204287; doi:10.3389/fcimb.2022.828605)

Table S1. Results of preliminary studies with targeted mutagenesis and nuclear localization

Intended mutation(s)	AR1	pos in AR1	AR2	pos in AR2	AR3	pos in AR3	AR4	pos in AR4	change in nuclear localization?
R56A (AR1)	R56A	13	0		L183I	6	0		no
G157A (AR2)	N57S	14	T162A	18	0		0		no
V190A (AR3)	N57S	14	0		V190A	13	0		no
M228R (AR4)	N57S	14	0		0		M228A	13	yes
R56A (AR1) and G157A (AR2)	R56A	13	T162A	18	0		0		yes
G157A (AR2) and V190A (AR3)	N57S	14	T162A	18	V190A	13	0		yes
V190A (AR3) and M228A (AR4)	0		T162A	18	V190A	13	0		yes

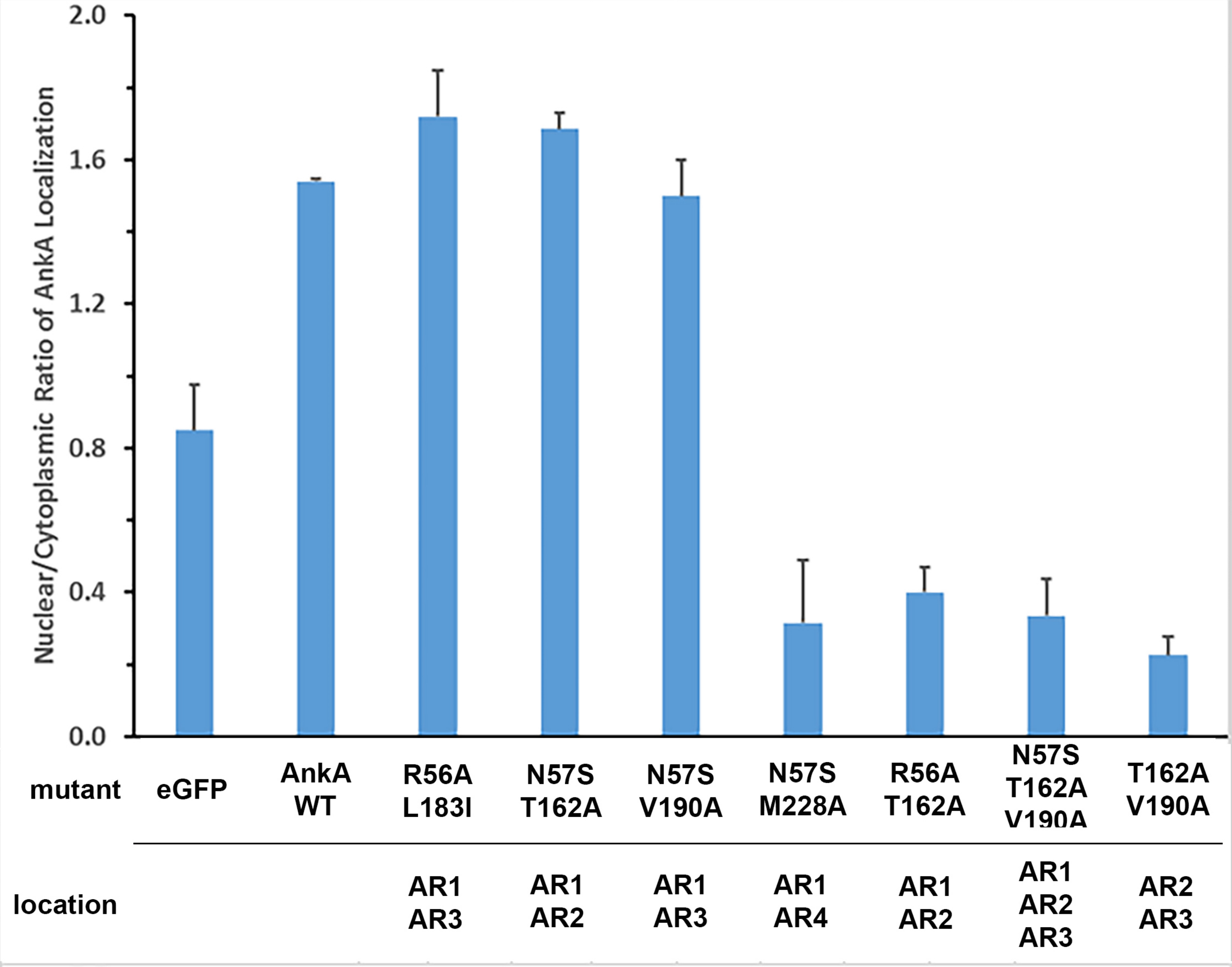

Supplement: Supplementary file 5 [file Table_1.pdf]
